# Supplementary material for: In vivo tracking of adenoviral-transduced iron oxide-labeled bone marrow-derived dendritic cells using magnetic particle imaging
Source: Eur Radiol Exp. 2023 Aug 15;7:42. doi: 10.1186/s41747-023-00359-4 (PMC10425309; doi:10.1186/s41747-023-00359-4)
Supplement: Supplementary file 1 — Additional file 1: Supplemental Table S1. Antibodies and reagents. Supplemental Fig. S1. Ad SPIO+ BMDC migrate to the popliteal lymph node following injection. One million Ad SPIO+ BMDC were fluorescently labeled with PKH26 immediately before hind footpad adoptive transfer. Two days later, excised popliteal lymph nodes were processed into 16 μm cryosections to observe PKH26 (a) and enhanced green fluorescent protein (b) fluorescence at × 100 magnification. Image inset outlined by a white square in (a and b) is shown at × 400 magnification for PKH26 (c) and eGFP fluorescence (d) as well as a PKH26 and eGFP overlay (e) to identify Ad SPIO+ BMDC in pLN. Scale bars = 100 µm (a-b) and 20 μm (c-e). Data is representative of n = 2 independent experiments with four mice per group. [file 41747_2023_359_MOESM1_ESM.pdf]

In vivo **tracking of adenoviral-transduced iron oxide-labeled bone marrow-derived dendritic cells using magnetic particle imaging**

ELECTRONIC SUPPLEMENTARY MATERIAL

Supplemental Table S1. Antibodies and reagents.

Target	Fluorochrome	Company	Clone
B220 (purified)	N/A	BD Biosciences (Mississauga, CAN)	RA3-6B2
CCR7	PE	Biolegend (San Diego, CA, USA)	4B12
CD11c	APC	Biolegend	N418
CD40	PE-Cy5	Biolegend	3/23
CD45	PE	Biolegend	30-F11
CD86	PerCP	Biolegend	GL-1
I-Ab	APC-Fire™ 750	Biolegend	AF6-120.1
I-Ab (purified)	N/A	Biolegend	25-9-17
ICOS Ligand	PE	Biolegend	HK5.3
OX40 Ligand	PE-Cy7	Biolegend	RM134L
TruStain FcX™	N/A	Biolegend	93
Cell proliferation dye	Tag-It Violet™	Biolegend	N/A
Fixable vital dye	Zombie NIR™	Biolegend	N/A
**Reagent**		**Company**	
2-Mercaptoethanol		Thermo Fisher Scientific (Burlington, CAN)	
Ad eGFP		Vector Biolabs (Malvern, PA, USA)	
BSA		Millipore Sigma (Burlington, CAN)	
CpG ODN 1826		InvivoGen (San Diego, CA, USA)	
FBS		Thermo Fisher Scientific	

FeREX®	Biopal Inc. (Worcester, MA, USA)
GM-CSF (recombinant)	PeproTech (Montreal, CAN)
HBSS	Thermo Fisher Scientific
Heparin sodium injection USP	Sandoz Canada Inc. (Boucherville, CAN)
HEPES (1 M)	Thermo Fisher Scientific
Histodenz™	Millipore Sigma
Interleukin- (IL)-1β	PeproTech
IL-4 (recombinant)	PeproTech
IL-6 (recombinant)	PeproTech
MEM non-essential amino acids solution (100X)	Thermo Fisher Scientific
PBS	Thermo Fisher Scientific
Penicillin-Streptomycin-L-Glutamine (100X)	Thermo Fisher Scientific
PKH26 red fluorescent cell membrane label	Millipore Sigma
Prostaglandin E2	Millipore Sigma
Protamine sulfate	Millipore Sigma
Rabbit complement (standard)	Cedarlane Labs (Burlington, CAN)
RPMI media	Thermo Fisher Scientific
Sodium pyruvate (100 mM)	Thermo Fisher Scientific
Synomag®-D	Micromod GmbH (Rostock, GER)
TNF-α (recombinant)	PeproTech

Ad Adenovirus, APC Allophycocyanin, *BSA* Bovine serum albumin, *Cy* cyanin, *eGFP* Enhanced green fluorescent protein, *FBS* Fetal bovine serum, *GM-CSF* Granulocyte-macrophage colony-stimulating factor, *HBSS* Hank's balanced salt solution, *HEPES* 4-(2-hydroxyethyl)-1-piperazineethanesulfonic acid, *ICOS* Inducible costimulator, *IL* Interleukin, *MEM* Minimal essential media, *PE* Phycoerythrin, *PerCP* Peridinin-chlorophyll-protein, *PBS* Phosphate-buffered saline, *RPMI media* Roswell Park Memorial Institute media, *TNF* Tumor necrosis factor.

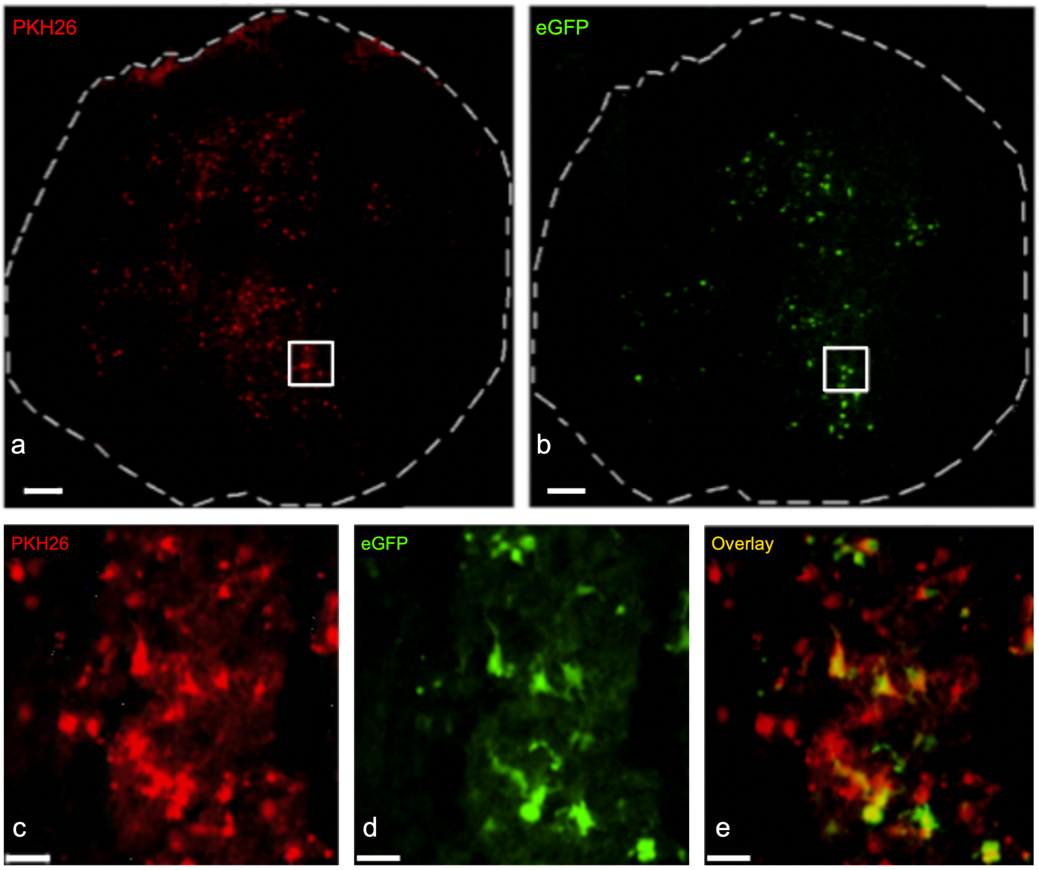

Supplemental Fig. S1. Ad SPIO⁺ BMDC migrate to the popliteal lymph node following injection. One million Ad SPIO$^+$ BMDC were fluorescently labeled with PKH26 immediately before hind footpad adoptive transfer. Two days later, excised popliteal lymph nodes (pLN) were processed into 16 µm cryosections to observe PKH26 (**a**) and enhanced green fluorescent protein (eGFP) fluorescence (**b**) at 10X magnification (scale bars = 100 µm). Image inset outlined by a white square in (**a**, **b**) is shown at 40X magnification for PKH26 (**c**) and eGFP fluorescence (**d**) as well as a PKH26 and eGFP overlay (**e**, yellow) to identify Ad SPIO$^+$ BMDC in pLN. Scale bars = 20 µm in (**c-e**). Data is representative of *n* = 2 independent experiments with four mice per group.
